# Examining Post COVID-19 Tourist Concerns Using Sentiment Analysis and Topic Modeling

**DOI:** 10.1007/978-3-030-65785-7_54

**Published:** 2020-11-28

**Authors:** Sreejith Balasubramanian, Supriya Kaitheri, Krishnadas Nanath, Sony Sreejith, Cody Morris Paris

**Affiliations:** 1 Department for Informatics, Technical University of Munich, Garching bei München, Bayern Germany; 2 Smart Tourism Education Platform (STEP) College of Hotel and Tourism Management, Kyung Hee University, Seoul, Korea (Republic of); 3 Department of Tourism and Service Management, MODUL University Vienna, Vienna, Wien Austria; Middlesex University Dubai, Dubai, UAE

**Keywords:** Tourism supply chain, Emotions, Lexicon-based approach

## Abstract

The COVID-19 pandemic has had a destructive effect on the tourism sector, especially on tourists’ fears and risk perceptions, and is likely to have a lasting impact on their intention to travel. Governments and businesses worldwide looking to revive and revamp their tourism sector, therefore, must first develop a critical understanding of tourist concerns starting from the dreaming/planning phase to booking, travel, stay, and experiencing. This formed the motivation of this study, which empirically examines the tourist sentiments and concerns across the tourism supply chain. Natural Language Processing (NLP) using sentiment analysis and Latent Dirichlet Allocation (LDA) approach was applied to analyze the semi-structured survey data collected from 72 respondents. Practitioners and policymakers could use the study findings to enable various support mechanisms for restoring tourist confidence and help them adjust to the’new normal.’

## Introduction

Travel and tourism are among the most affected sectors due to the worldwide outbreak of the COVID-19 pandemic. According to United Nations World Tourism Organization (UNWTO), the sector which witnessed an unprecedented decline of around 98% in international tourists in May 2020, compared to the same time last year, is likely to face a drop of up to 1.1 billion international tourists and US$ 1.2 trillion in revenues in 2020, putting 100 to 120 million jobs at risk [1]. Many countries, especially those most reliant on travel and tourism revenue, are implementing a wide range of measures for the reopening of the tourism economy and stimulate the recovery of the sector [2]. However, considerable challenges remain ahead, starting with the unknown duration of the pandemic itself and the global economic recession.

While immediate measures such as lifting travel restrictions, adjusting or simplifying visas requirements, and cutting tourist taxes could support the sector in the short term, for the long-term recovery, it is critical to restore consumer confidence and rebuild demand. This is because, although the COVID-19 started as a physical health crisis, there is growing evidence that COVID-19 is having a profound detrimental effect on the mental health of the general population, including tourists, which needs to be addressed urgently [3, 4]. The impact of the pandemic on people’s mental health, especially fear of falling ill and dying, fear of infecting others, anger, and anxiety are extremely concerning and may have a far more significant and lasting impact on the tourists’ intention to travel. Hence, we must develop a critical understanding of tourist sentiments and concerns across the supply chain so that various support mechanisms can be put in place for restoring tourist confidence and rebuilding demand.

This formed the motivation of this study, which aims to examine tourists’ sentiments and concerns across the tourism supply chain starting from the dreaming/planning phase to booking, travel, stay, experiencing, and departure. The specific objectives are as follows:

1. To understand the emotions related to tourist concerns through sentiment analysis.
2. To identify common topics of concern for the different stages in the tourism supply chain using Latent Dirichlet Allocation (LDA) approach.

In the next section, the research methodology adopted in this study is detailed. The findings are discussed in section three, with the final/concluding section covering the implications and suggestions for future research.

## Research Methodology

**Fig. 1.**
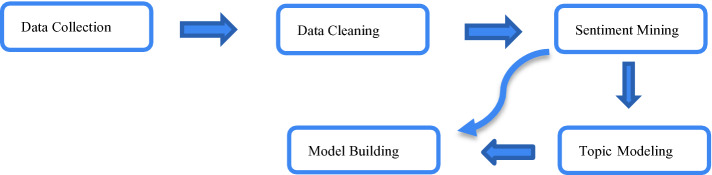
Research methodology

The primary data was collected from participants belonging to different nationalities using qualitative, semi-structured surveys (an ideal tool for exploratory research) using Qualtrics. The survey link was sent to participants via email. The questions focused on capturing tourists’ COVID-19 concerns during the phases of the tourist supply chain. The final data set for analysis included 72 responses from participants from nine countries, of which 64% was male, and 36% was female. In line with our research objectives, Natural Language Processing (NLP) using sentiment analysis and the LDA approach was applied for analyzing tourist concerns across the different phases of the tourism supply chain. Previous studies have shown that applying both sentimental analysis and LDA on the dataset provides rich and meaningful insights on public opinion [5]. Figure 1 explains the research methodology and its procedure.

The survey data was first cleaned to remove stop words, meaningless characters such as HTML tags, punctuation, numbers, and emoticons. It is important to understand the tourist concerns and their associated emotions. Sentiment analysis can help us understand the polarity (negative or positive) or the extent of emotions (joy, anger, and others) in a language, and it was used to uncover the various hidden emotions (related to concerns) of tourists [6, 7]. A lexicon-based approach, which involves calculating the sentiment from the semantic orientation of words or phrases that occur in a text, was used for sentiment analysis [8]. Each response for the questions in the survey was considered as a document in the dataset, and then analysis was performed for each of those responses. The lexicon-based emotion analysis package “syuzhet”- which is available in R (Version 3.6.2), was used for identifying the emotions contained in each response. Although both positive (e.g., trust, joy) and negative emotions (e.g., anger, fear) could be computed, given the focus of this study was to explore the concerns expressed by the tourists, only negative emotions were considered. The count of specific emotions in a comment was used for the analysis.

The next objective was to uncover the distinct themes/reasons in the tourist con-cerns. Topic modeling encompasses different algorithms that process text data to identify dominant themes based on the similarity of co-occurrences of the words [7, 9]. This research uses the Latent Dirichlet allocation (LDA) algorithm for topic modeling because of its ability to control the number of words and topics so that an empirical model can be developed. One of the prime objectives of this exercise was to understand if there are a few dominant topics in the dataset corresponding to each of the travel phases. Microsoft Azure Machine Learning Studio software was used for the LDA analysis.

## Results and Discussions

This section discusses the results of sentiment analysis and topic modeling.

### Tourist Emotions (Sentiment Analysis)

**Fig. 2.**
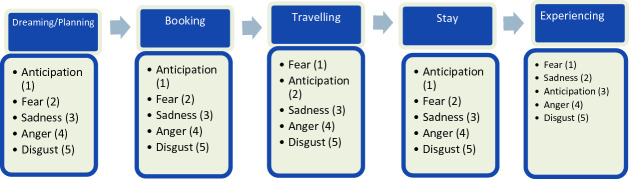
Results of sentiment analysis

The five negative emotions, namely anticipation of uncertain/undesirable events, fear, sadness, anger, and disgust hidden in the tourist concerns related to different phases of the tourist supply chain was computed and ranked. The rank of emotions across each supply chain stage is given in Fig. 2.

As seen in the figure, the emotions related to anticipation of uncertain/undesirable events (ranked 1^st^ in dreaming, booking, and stay phase) and fear (ranked 1^st^ in traveling and experiencing stage) dominate the tourist concerns across the different stages of travel. The anticipation of uncertain/undesirable events was mostly attributed to the dynamic changes in the travel rules and regulations in different countries, such as changes in quarantine rules. In the words of one of the respondents, *“Changes in quarantine restrictions to and from the destination is a concern.”* Similarly, in the words of another participant, *“I would never book without being fully sure that I could travel. Will the country remain open? Will I be able to return home?”* Emotion of fear was mostly related to the fear of getting infected/re-infected and infecting others. People are mainly worried about their safety and that of their loved ones. In the words of a participant, *“Fear of catching infections at the airport as it is the main hub where you tend to be around people from different parts of the world.”* This is mostly in line with the findings of World Economic Forum [10].

### Key Tourist Concerns (Topic Modeling)

Table 1 presents the topic modeling results based on the bi-gram analysis under LDA.

**Table 1.** Topic modeling results.

Travel Stage	Topic 1	Topic 2	Topic 3
Dreaming/Planning	Planning with restrictions	Technological Assistance during COVID Travel (Quarantine, Tracking, etc.)	–
Booking	Cancellation and refund concerns	Availability of convenient flights and hotels	–
Traveling	Security and Safety	COVID protocols	Fear of Getting Infected
Staying	COVID Hygiene	Access to Facilities nearby	–
Experiencing	COVID Situation at the Destination	Weather and Health Facility	–

Planning to travel while restrictions are still in place and relevance of technology (or lack thereof) such as smart COVID-19 applications such as contact tracing to detect nearby cases emerged as the two distinct concerns during the dreaming/planning phase. The latter further reiterates the significance of technological advances in improving tourist experiences. Cancellation and refund issues and availability of flights to destinations and hotels are the main concerns that worry people in the booking phase. For the travel and stay phase, the majority of the concerns are related to COVID protocols, hygiene, health & safety, availability of good accommodation, restaurants, COVID testing facility, PPE kit in flight, etc. For instance, one of the participants mentioned the following concern *“How good is the hotel sanitation and cleanliness. How trained is the staff at the hotel to deal with COVID19 related issues?”* The concerns in the experiencing phase are mainly related to the destination itself, such as the social distancing measures in place, the number of active cases in the destination, and the availability of decent healthcare facilities in case of any infection. In the words of one of the respondents, “*Reaching the location, I would concern about the measures the country employs to prevent the spread of the virus and how strict the people in that country are aware of and comply with those measures*.” Some of these tourist concerns topics identified are similar to the ones specified in the recent COVID-19 tourism literature [11, 12].

## Conclusion

The preliminary findings of this study provides insights into the tourists’ post-COVID-19 travel concerns. Given the heightened uncertainty in the tourism sector due to the pandemic, the study provides valuable insights for practitioners and policymakers to gauge better and manage tourist confidence levels, perception of travel as a risk, and any changes in their preference and behavior. Moreover, the study is timely as countries are faced with challenges of containing the second or third wave of spread and, at the same time, manage the reopening of the tourism economy [2]. Primary research on tourist sentiment analysis and modeling the tourist concerns during COVID-19 could prove to be an important tool in both assessing and monitoring the recovery of the industry. However, given the early stages of the paper, there are limitations. The small sample size of the study limits the generalizability of the findings. Also, the concerns were understood only from tourist perspectives, and concerns of other stakeholders were not considered. Future studies could adopt a multi-stakeholder approach. Further, future research could attempt triangulating the primary findings using secondary data such as from travel websites and social media.
